# Cytochromes P450, Oxygen, and Evolution

**DOI:** 10.1100/tsw.2001.22

**Published:** 2001-04-19

**Authors:** David F.V. Lewis, Grainné Sheridan

**Affiliations:** School of Biomedical and Life Sciences, University of Surrey, Guildford, Surrey, GU2 7XH, UK

**Keywords:** cytochrome P450, oxygen, free radicals, evolution, oxidative metabolism, reactive oxygen species

## Abstract

The role of the cytochrome P450 superfamily of heme-thiolate enzymes in oxidative metabolism is discussed in the context of evolutionary development. Concordances between the rise in atmospheric oxygen content, elaboration of the P450 phylogenetic tree and the accepted timescale for the emergence of animal phyla are described. The unique ability of the P450 monooxygenase system to activate molecular oxygen via the consecutive input of two reducing equivalents is explored, such that the possibility of oxygen radical generation and its toxic consequences can be explained in mechanistic terms, together with an appreciation of the ways in which this oxygen activating ability has been utilized by evolving biological systems in their adaptation to an increasing atmospheric oxygen concentration over the past two billon years.

## INTRODUCTION

The cytochromes P450 (CYP) constitute a superfamily of enzymes, present in every biological kingdom[1], which are involved in the oxidative metabolism of a vast number of chemicals, both endogenous and exogenous, encompassing diverse structural classes and functionality of catalytic reactions[2,3].

P450s are now classified as heme-thiolate enzymes[4] rather than cytochromes which are redox components in various cellular electron transfer pathways, such as the cytochromes a, b, and c, although all of these enzymes are hemoproteins because they all contain a heme prosthetic group. The redox potentials of hemoproteins vary considerably due to the influence of the protein environment and the particular heme ligands involved[5]. This is illustrated in Table 1 where the iron redox potentials (E° values) of various hemoproteins are compared. It can be appreciated from Table 1 that P450s possess relatively high negative iron redox potentials in comparison with other hemoproteins. This is due to the characteristic cysteine ligand in P450 and also to the significant amount of heme exposed to the environment at the distal face where, in the absence of substrate, there is a bound water molecule coordinating the iron by occupying the distal heme site[6]. The binding of a substrate to P450 has a profound effect on the redox potential, together with other physicochemical characteristics, which triggers the entire catalytic cycle of the enzyme (*vide infra*).

As far as enzymes in general are concerned, however, the P450s can be regarded as being exceptional in their phenomenal substrate diversity, because most enzymes usually only accept substrates from a well-defined structural class[7]. In order to appreciate why this is so, it will be necessary to consider, first of all, the development of the P450 superfamily in the context of biological evolution over the past 3500 million years.

## P450 ENZYMES AND EVOLUTION

Over 1200 individual P450s have had their protein sequences determined to date[1] and these are classified by gene family and subfamily using a numerical system to designate the P450 family, together with an alphanumeric symbol for the subfamily and individual protein. This system of classification is based on percentage sequence homology between P450s and, therefore, facilitates the construction of a phylogenetic tree for the enzyme superfamily which can be related to the development of biological species over time on the basis of specific mutation rates for the P450 genes[8,9,10]. Fig. 1 shows that the evolution of the P450 superfamily mirrors the generally ac-

**TABLE 1**
**Iron Redox Potentials for Hemoproteins**

| Hemoprotein | % Heme Exposure | $E^0_{Fe^{3+}/Fe^{2+}}$ (mV) | Heme Ligands |
|---|---|---|---|
| Cytochrome $c_2$ | 6 | 320 | Met, His |
| Cytochrome c | 4 | 260 | Met, His |
| Cytochrome $c_{550}$ | 5 | 250 | Met, His |
| Hemoglobin α | 14 | 113 | His, His |
| Hemoglobin β | 20 | 53 | His, His |
| Myoglobin | 18 | 47 | His, His |
| Cytochrome $b_5$ | 23 | 20 | His, His |
| Cytochrome P450 | ~40 | -400 | Cys, $H_2O$ |

*Abbreviations:* Met = methionine; His = histidine; Cys = cysteine.

*Note:* The relationship between redox potential ($E^o$) and % heme exposure to the aqueous environment is as follows:

$$E^o_{Fe^{3+}/Fe^{2+}} \text{ (mV)} = 14.94\% \text{ exposure} + 343.88 \quad n = 7;\ s = 37.36;\ R = 0.96;\ F = 46.0 \quad (\pm 1.95)$$

This expression was derived for the first 7 proteins in the above table using the data reported by Stellwagen[78] and this was used to estimate the % heme exposure in P450[2]. The redox potentials in P450s vary depending on the isoform and whether there is a bound substrate, which lowers the redox potential. For example, camphor binding to $P450_{cam}$ (CYP101) is accompanied by a change in $E^o$ from -313 to -173 mV[52]. According to the above equation, substrate binding to $P450_{cam}$ can be expected to reduce the heme exposure from 44 to 35%[2].

cepted timescale for the development of life on Earth[2,11], whereas Fig. 2 provides an example of a phylogenetic tree for selected P450s in various animal species using the UPGMA* approach (see legend to Fig. 2 for details). There are certain correspondences (see Table 2) between specific evolutionary events and the elaboration of the P450 phylogenetic tree which support this viewpoint[2,8]. In particular, it is now currently accepted that around 2,000 million years ago the earth's atmosphere started to become oxygenated[12], leading to the emergence of eukaryotic species distinct from the original prokaryotic biota[13]. Although it is thought that the ancestral P450 gene may have arisen in a prokaryotic species about 3,500 million years ago[14] when the terrestrial atmosphere was reducing in nature, there is likely to have been sufficient oxygen available for P450-mediated catalytic transformations of organic carbon sources in bacterial systems. However, with the advent of free oxygen in the atmosphere[15,16] produced from photosynthetic bacteria, unicellular eukaryotic species developed by the fusion of prokaryotes (which became cell organelles) and their encapsulation in a protective cell membrane[17]. It is possible that the increased oxygen concentration may have been the driving force for this major biological development[18], because the formation of eukaryotic organisms would have afforded some protection from the potentially damaging effects of reactive oxygen species (ROS). Indeed, the development of enzyme systems such as catalase and superoxide dismutase[19] to deal with ROS, like peroxide and superoxide, would have enabled the catalytic functions of P450s to diversify without the cytotoxic side-effects of uncoupled dioxygen reduction. Consequently, increasing oxygenation of the atmosphere facilitated the evolution of specific P450 isozymes which could be 'tailored' to recognize certain structural classes of chemicals that possessed the potential for important endogenous roles in the newly emerging eukaryotes[11].

It is generally accepted that by 1000 million years ago animal-plant divergence had occurred together with the development of sexual reproduction[17]. It is possible that branching of the eukaryote stem of the P450 phylogenetic tree may have taken place during this period, where bifurcation into mitochondrial and microsomal P450s would have coincided with the requirement for steroid hormone biosynthesis from cholesterol in order to regulate sexual characteristics[3]. However, the situation is likely to have been more complex than is

* Unweighted Pair Group Method of Analysis.

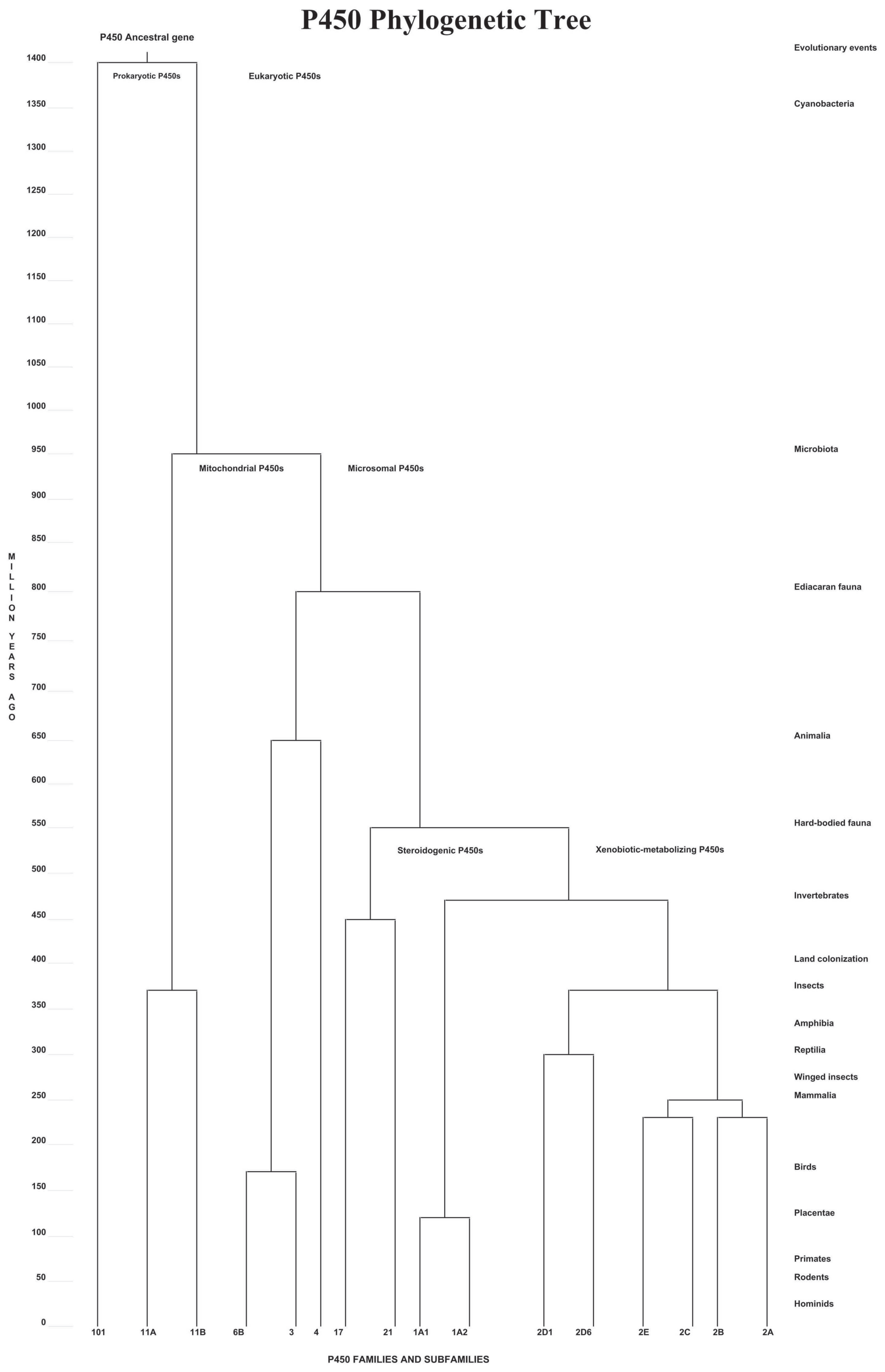

**FIGURE 1.** An abbreviated version of the P450 phylogenetic tree (adapted from References 2 and 11) using data compiled from a number of sources (see text for details).

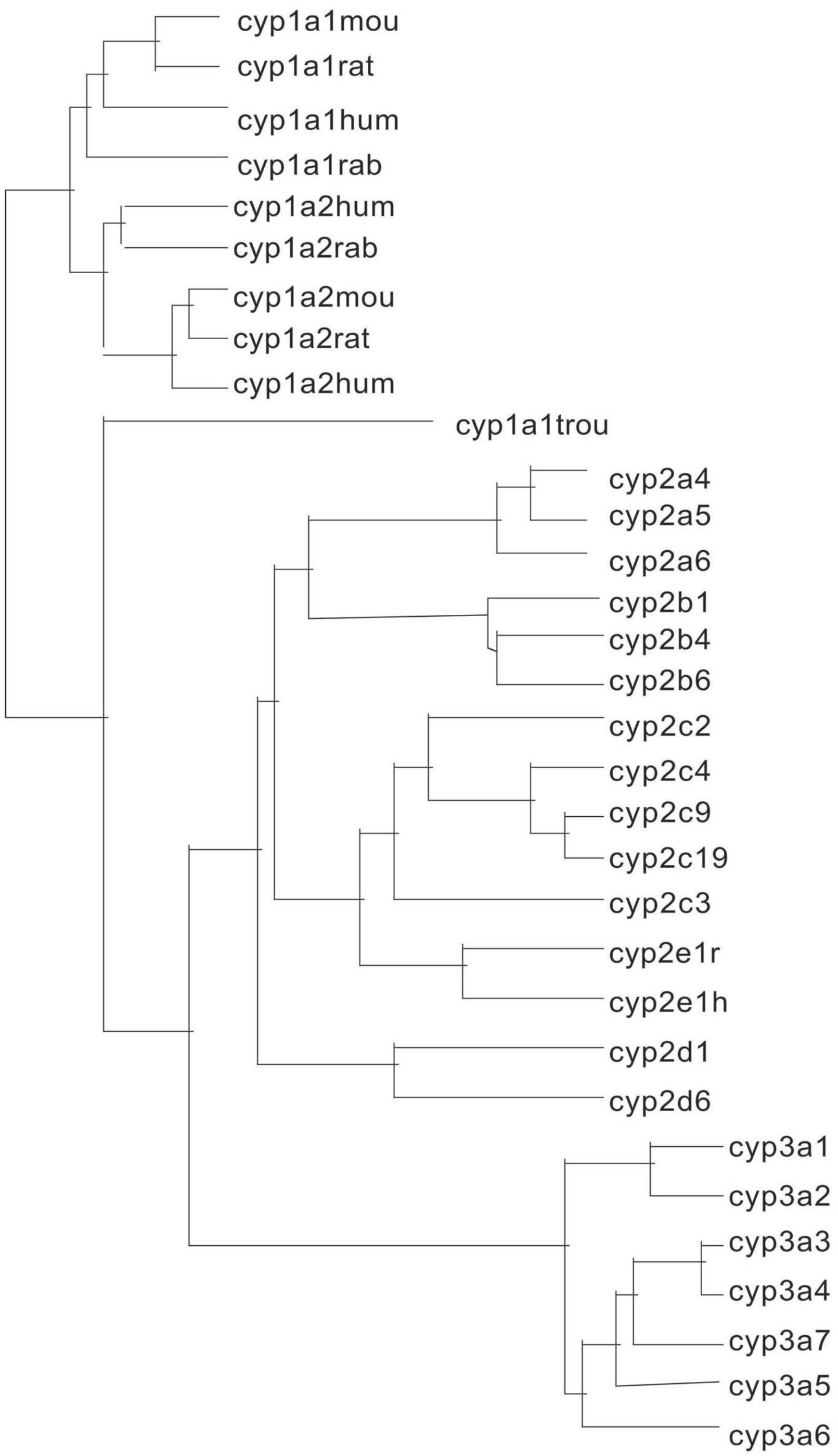

**FIGURE 2.** Phylogenetic tree of cytochrome P450 protein sequences from families CYP1, CYP2 and CYP3 inferred using the UPGMA tree drawing algorithm with Kimura calculated distances. Protein sequences were aligned using ClustalW with manual readjustments based on known secondary structure conformation. Alignments of P450 protein sequences have been reported previously[2,11].

**TABLE 2**
**Concordances between Evolution and P450 Development**

| MYA | Biological Development | P450 Superfamily Divergences |
|---|---|---|
| 2000 | Eukaryotic cells | Prokaryotic-Eukaryotic CYPs |
| 1000 | Plant-animal divergence | Mitochondrial-Microsomal CYPs |
| 800 | Ediacaran biota | CYP3 and 4 split from other microsomal CYPs |
| 650 | Divergence of animalia | CYP3 and CYP4 divergence |
| 550 | Animal phyla radiate | CYP1 and 2 diverge from CYP17 and 21 |
| 470 | Invertebrates | CYP1 and CYP2 diverge |
| 450 | Land colonization | CYP17 and CYP21 diverge |
| 370 | Amphibia | CYP2D diverges from CYP2 |
| 300 | Bird-mammal divergence | CYP2D radiates |
| 250 | Reptilia | Divergence of CYP2 family |
| 230 | Mammalia | CYP2A and CYP2B diverge, CYP2C and CYP2E diverge |
| 120 | Placental mammals | CYP1A1 and CYP1A2 diverge |
| 80 | Mammalian radiation | Speciation of P450 families and subfamilies |

MYA = million years ago
Adapted from Reference 11.

apparent from phylogenetic comparisons because of the probable prokaryotic origins of eukaryotic cell organelles, such as mitochondria[20]. Moreover, there is evidence to suggest that animal species may have increased their body size in order to minimize the mutagenic effects of ROS[21] with mitochondria representing the major organelle associated with oxygen consumption.

Elaboration of the animal phylogeny around 800 million years ago, as evidenced by the Ediacaran biota fossil data, appears to correspond with a further branching of the P450 tree where the CYP3 and CYP4 families split from the main microsomal P450 limb[22]. The functions of these P450s lie in steroid and fatty acid metabolism, respectively, and as such play important roles in the regulation of steroid hormones, prostaglandins and poly-unsaturated fatty acids, such as arachidonic acid, for example[23,24].

The steady increase in atmospheric oxygen levels, paralleling the general rise in animal species over the last 2 billion years[25,26], should be set, however, against a backdrop of terrestrial cycles which probably contributed to extinction events over geological time[27]. Radiation of the animal phyla at around 550 million years ago, during what is termed the Cambrian ‘explosion’, followed the divergence of animalia about 650 million years ago; concordances with the P450 tree development indicate that, initially, the CYP3 and CYP4 families separated, with the divergence of CYP1 and CYP2 from the remaining steroidogenic P450 families, CYP17 and CYP21, occurring in the Cambrian period[22]. In this way, it would appear that the P450s primarily associated with exogenous metabolism (CYP1, CYP2 and CYP3) separated from those involved in endogenous compound metabolism (CYP4, CYP11, CYP17 and CYP21) over a period of 500 million years, from 1000 million years ago to about 500 million years ago, at a time (termed the Neoproterozoic era) where animal species underwent significant evolutionary development[27].

In addition to the concurrent rise in the oxygen content of the atmosphere, it is possible that global plate tectonic changes, such as opening of the Iapetus ocean (circa 700 million years ago) may have played a part in biological evolution by contributing to major extinction events[28]. In fact, there is some degree of correlation between the supercontinental cycle and disappearance of certain species, via its effect on long-term eustatic sea level fluctuations[29]. This is also apparent in the Mesozoic and Cenozoic eras, where it has been argued[30] that continental fission and fusion may have led to the ordinal divergence of avian and mammalian species.

However, a major concordance between the microsomal P450 branching and animal evolutionary development has been documented[8] which corresponds to the Devonian period when animals began to colonize land[27]. By an example of coevolution which has been termed animal-plant ‘warfare’, it would appear that animal species developed specific xenobiotic-metabolizing P450s to detoxify poisonous plant products which had been biosynthesized by various plant

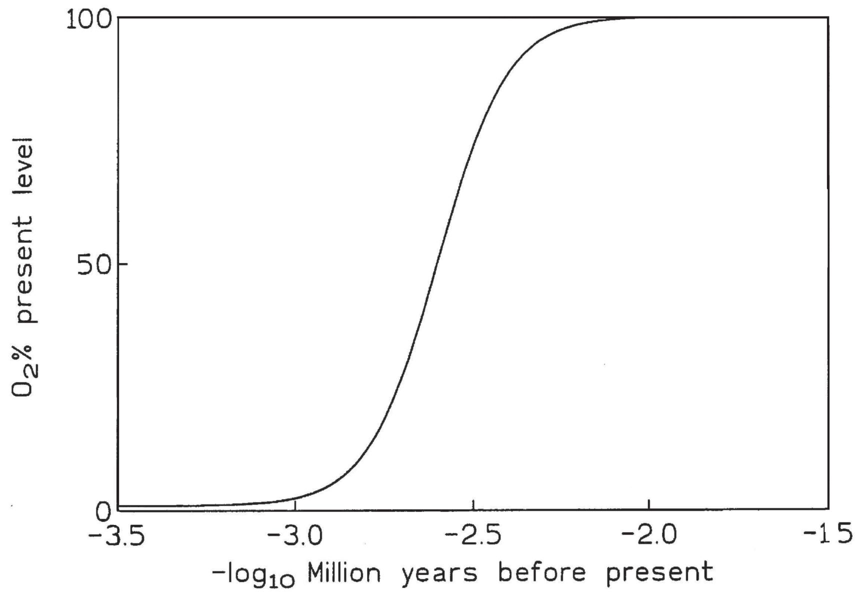

**FIGURE 3.** Increase in atmospheric oxygenation over the past 3,500 million years plotted on a logarithmic scale (adapted from Reference 2 and based on data compiled by Cloud[12]).

species to deter animal predators[8]. It is interesting to note that the Devonian period corresponds to the time when the oxygen content of the atmosphere rose dramatically, probably reaching about 50% of the present level around 397 million years ago[12]. Fig. 3 shows that if the relevant data are plotted using a logarithmic time axis, the increase in percentage oxygen concentration of the atmosphere exhibits a sigmoidal relationship over a 2000 million year period[2,11].

According to estimations based on sequence Homology and generally accepted mutation rates, the divergence of CYP1 from CYP2 took place around 470 million years ago during the development of invertebrates, whereas the emergence of amphibia and land colonization (about 400 million years before present) corresponds with initial branching of the CYP2 family where CYP2D divergence may have represented a response to the evolutionary pressures posed by toxic plant products[11]. As the preferred substrates of CYP2D possess basic nitrogen atoms, and many of these are ligands (antagonists or agonists) of G-protein–coupled receptors associated with the transduction of neurological signals, it seems likely that initial plant toxins may have acted on the central nervous systems of predatory species who, in turn, would have required an enzyme system capable of detoxifying such hazardous chemicals. Consequently, it is possible to rationalize that the first bifurcation of the CYP2 family probably would have been that of the CYP2D subfamily, as indicated by sequence comparisons[8].

The generally accepted times for the divergence of avian and mammalian species at about 300 million years ago, radiation of reptiles around 250 million years ago, and further mammalian development 230 million years before present correspond with the divergence of the CYP2 family into the five major subfamilies, CYP2A through to CYP2E[31]. This significant elaboration of the CYP2 family may represent an evolutionary response to other plant toxins, such as flavonoids and alkaloids, although there is emerging evidence for endogenous roles of various CYP2 subfamilies. For example, enzymes of the CYP2A, CYP2B, and CYP2C subfamilies exhibit significant sex differences in steroid metabolism for experimental rodent species, such that these may have a function in regulating steroid hormone levels[32]. Moreover, the CYP2D and CYP2E subfamilies have been implicated in neurotransmitter metabolism[33] and in gluconeogenesis, respectively[34].

Placental mammals developed around 120 million years ago; this coincides with the likely divergence between two related P450s in the CYP1 family, namely, CYP1A1 and CYP1A2[35]. It is possible that the CYP1A2 isozyme is required for the regulation of oestrus, as it has been established[36] that CYP1A2 levels are modulated by the menstrual cycle. Consequently, placental mammals would have presumably required

CYP1A2 to vary the levels of oestrogens at certain stages in the female monthly cycle. By 80 million years ago, the mammalian radiation is coincident[11] with extensive speciation of mammalian P450 genes, which are likely to have formed orthologous proteins during this period[37] when most modern mammalian species evolved, including primates[38].

One of the intriguing aspects of primate development in the context of P450 evolution lies in the finding that there are substantial differences in the hepatic P450 complement of New World and Old World primate species. Essentially, the New World monkeys possess significant levels of CYP1A2 but little or no CYP2A6, whereas the opposite is found for Old World primates[39]. *Homo sapiens*, however, possesses both CYP1A2 and CYP2A6 in approximately equal amounts. The reasons for these changes may lie in continental drift followed by altered dietary habits as a consequence of different habitats[40]. It is possible that, during the time of separation between the South American and African continents over 100 million years ago, two pro-simian populations became geographically isolated such that these effectively separated species evolved differently as a response to habitat and food sources[11]. This would have led to substantial differences between CYP1A2 and CYP2A6 levels in New and Old World primates because the former have omnivorous dietary habits, whereas the latter are essentially herbivores[41]. In this respect, it is important to recognize that CYP2A6 metabolizes many plant flavonoids and CYP1A2 is involved in the oxidative metabolism of nitrogenous compounds, such as heterocyclic amines and amides[42]. Mankind may have originally been herbivorous but, at some stage in their development, became more carnivorous, with the consumption of cooked meat representing a relatively recent dietary practice[43]. It is logical, therefore, that *Homo sapiens* now possesses roughly equivalent percentages of CYP1A2 and CYP2A6, which would be consistent with our current dietary preferences.

As far as drug metabolism, in particular, is concerned, it has been well established that human ethnogeographical populations exhibit genetic polymorphisms towards certain classes of drug substrates, many of which correspond to point mutations in P450 genes[44]. Consequently, small percentages of different human populations show impaired metabolic competence for an increasing number of pharmaceutical agents which are either CYP2D6 or CYP2C19 specific[42]. The reasons for these genetic polymorphisms are, as yet, unknown but may be a natural consequence of the genetic diversity encountered in human populations[8] possibly associated with dietary differences[44], whereas other animal species tend to be more genetically pure, although the strain differences encountered in some experimental rodent species could represent an analogy with the known genetic polymorphism exhibited by human populations.

A possible reason for mankind possessing a complement of P450 isozymes capable of metabolizing the majority of known pharmaceutical agents may lie in the elaborations of the P450 phylogenetic tree which took place over the last 400 million years[11]. Most modern drugs have been designed on templates afforded by existing plant products, and this is also probably true of the various agrochemicals to which we are exposed. Consequently, *Homo sapiens* would be able to utilize the P450 system of xenobiotic metabolizing enzymes in the oxidative metabolism of pharmaceutical agents in current use, despite the fact that these isozymes had previously been developed to detoxify plant chemicals[8]. In order to understand the way in which P450s are able to metabolize such a wide variety of chemicals, it is important to consider their properties and the typical P450 enzyme's catalytic cycle.

## PROPERTIES AND CHARACTERISTICS OF P450S AND THEIR SUBSTRATES

The average molecular mass of P450 enzymes is around 50kDa and the protein consists of polypeptide chain of about 500 amino acid residues (reviewed in Reference 2). All P450s contain a heme prosthetic group with a thiolate fifth ligand from an invariant cysteine residue (proximal to the heme face) which forms part of a high conserved 10-residue signature motif identifiable in every P450 sequence known[1]. The heme moiety, with its cysteine thiolate ligand, is able to bind (and, subsequently, activate) oxygen by splitting the diatonic molecule into water (via protonation) and a bare oxygen atom[4]. This highly reactive single oxygen atom combines with a bound organic substrate which is held in position by key amino acid residues within the enzyme's active site at the distal heme face, such that oxygenation at a specific region of the substrate molecule occurs[4]. Due to the fact that a single oxygen is inserted into the substrate, the P450 enzyme is sometimes referred to as a mono-oxygenase, and the mono-oxygenated product (i.e., metabolite) of the reaction is usually a hydroxy compound (alcohol or phenol), epoxide, N-oxide (or S-oxide), or a hydroxylamine[45], although other metabolites are known[46,47]. For a hydrocarbon substrate, RH, it is possible to represent the general P450-catalyzed reaction[3] as follows:

$$\underset{\text{substrate}}{RH} + O_2 \xrightarrow[2E^-]{2H^+} \underset{\text{metabolite}}{ROH} + H_2O$$

where the two reducing equivalents ($2H^+$, $2e^-$) are supplied by NADPH (or NADH) and the reduction is mediated by either an FAD-containing flavoprotein and iron-sulphur redoxin or an FAD- and FMN-containing flavoprotein oxidoreductase, depending on the type of P450 system[48].

The substrates of P450 isozymes, which may be in excess of 200,000 chemicals[46], comprise virtually every class of organic compound, including polyaromatic hydrocarbons (e.g., benzo(a)pyrene), steroids (e.g., testosterone), nitrosamines (e.g., DMN), alkenes (e.g., butadiene), ketones (e.g., acetone), alcohols (e.g., ethanol), nitro compounds (e.g., p-nitrophenol), amides (e.g., 2-acetylaminofluorene), barbiturates (e.g., phenobarbital), heterocyclic compounds (e.g., caffeine), halothanes (e.g., chloroform), alkaloids (e.g., nicotine), ethers (e.g., aflatoxin), organophosphates (e.g., parathion), amines (e.g., benzphetamine), carboxylic acids (e.g., lauric acid), organochlorine pesticides (e.g., DDT), azo compounds (e.g., sulphasalazine), esters (e.g., MEHP), benzodiazepines (e.g., diazepam) and ß-blockers (e.g., propranolol). Table 3 summarizes the substrate specificity exhibited, for example, by human liver P450s[42].

Due to the enormous structural diversity of their substrates (in stark contrast with those of other enzymes), P450s are sometimes referred to as mixed function oxidases because of the extraordinary variety of functional groups which may be present in their substrate molecules[4]. The reasons for such a vast number of potential substrate entities lie in the relatively large number of P450 enzymes, even within a single species, and the distinct pattern of amino acid residues in the active sites of some P450s which enable contact with many different kinds of substrates[2]. There is, however, an important distinction between those P450s that are involved in endogenous compound metabolism and those which metabolize xenobiotic chemicals. In general, the P450s responsible for the metabolism of endogenous substrates such as steroids, prostaglandins, eicosanoids, and fatty acids possess a well-defined substrate specificity for each enzyme which, in some cases, is specific for one compound only[3]. On the other hand, those P450s which play a major role in the metabolism of xenobiotics exhibit relatively wide substrate specificities, although there are certain structural determinants that are able to define whether a given chemical is likely to be a substrate for one P450 or another[49]. There are also many examples where a chemical is metabolized by more than one P450, but at different sites on the substrate molecule; as is the case with the anti-ulcer drug omeprazole, which is metabolized by both CYP3A4 (S-oxidation) and CYP2C19 (5-methyl hydroxylation) in human liver[42].

For P450s that metabolize foreign chemicals, it is possible for enzymic activation to occur leading to the formation of reactive intermediates[47] which are potentially toxic; some P450s have been implicated in the metabolic activation of many known carcinogens such as benzene, benzo(a)pyrene, ß-naphthylamine, cyclophosphamide, butadiene, dialkylnitrosamines, aflatoxin, and several heterocyclic amines formed during the pyrolysis of meat proteins[50]. In fact, most procarcinogen activation is mediated by P450s and, primarily, it appears that enzymes of the CYP1A and CYP2E subfamilies are mainly responsible for the production of genotoxic reactive species in both experimental animals and man, following the exposure/ingestion of potentially carcinogenic chemicals[3]. However, the remaining xenobiotic-metabolizing P450s in mammalian liver are generally associated with the detoxica-

**TABLE 3**
**Human Xenobiotic-Metabolizing P450s: Substrates, Inducers, and Inhibitors[42]**

| CYP | Substrate | Specific Reaction | Inhibitors | Inducers |
|---|---|---|---|---|
| 1A1 | 7-Ethoxyresorufin | O-Deethylation | 9-Hydroxyellipticine | TCDD |
| 1A2 | Caffeine | $N_3$-Demethylation | Furaphylline | Heterocyclic amines |
| 1B1 | 17β-Estradiol | 4-Hydroxylation | Propofol | Indolocarbazole |
| 2A6 | Coumarin | 7-Hydroxylation | Pilocarpine | Dexamethasone |
| 2B6 | 7-Ethoxy 4-trifluoro-methyl coumarin | O-Deethylation | Orphenadrine | Phenobarbital |
| 2C9 | Tolbutamide | 4-Methyl hydroxylation | Sulfaphenazole | Barbiturates |
| 2C19 | Omeprazole | 5-Methyl hydroxylation | Fluconazole | Barbiturates |
| 2D6 | Debrisoquine | 4′-Hydroxylation | Quinidine | None known |
| 2E1 | 4-Nitrophenol | 2-Hydroxylation | 4-Methyl pyrazole | Ethanol |
| 3A4 | Nifedipine | N-Oxidation | Ketoconazole | Dexamethasone |

TCDD = 2,3,7,8-tetrachlorodibenzo-p-dioxin.

tion of exogenous compounds[50]. Consequently, P450s can be regarded as a 'double-edged sword' with respect to the metabolism of foreign chemicals, being able to detoxify or activate depending on the nature of compound and the type of P450 involved in oxygenating the substrate molecule. To explore this aspect further, it will be necessary to review the operation of P450's catalytic pathway and consider the way in which oxygen is activated.

## THE P450 CATALYTIC CYCLE AND OXYGEN ACTIVATION

The proximal sulphur ligand from an invariant cysteine residue in its thiolate state brings about activation of the heme-bound oxygen molecule, which binds to P450 at the distal heme face[51]. Other hemoproteins, such as hemoglobin and myoglobin, are able to bind oxygen similarly to that of P450, but activation does not occur because these oxygen binding carrier proteins possess a proximal histidine residue instead of cysteinate as encountered in P450[51]. The difference in effect between the oxygen carrier proteins and P450 is related to the $Fe^{3+}/Fe^{2+}$ redox potential of the heme iron which, in the case of P450, is substantially more negative than other hemoproteins[5]. Moreover, substrate binding to P450 has a marked effect on the iron redox potential, together with producing a significant modulation of iron spin-state equilibrium[52].

The P450 reaction involves a controlled activation of oxygen by the consecutive input of two electrons from NADPH (or NADH) via intermediary redox partners, which differ between mitochondrial, microsomal, and bacterial P450 systems[48]. Most bacterial P450s (except some of those in the CYP102 family) utilize an iron sulphur redoxin to mediate electron transfer from an NADH-dependent FAD-containing flavoprotein reductase[53]. Mitochondrial P450s in animals resemble the bacterial systems in containing an electron transport chain consisting of an NADH-specific FAD-dependent oxidoreductase and an iron sulphur redoxin which transfers electrons to P450[48]. This striking similarity between prokaryotic and animal mitochondrial P450 systems adds weight to the view that mitochondria may have originated from bacterial species[54,55]. In contrast, microsomal P450s utilize an FAD- and FMN-containing oxidoreductase flavoprotein for transferring electrons from NADPH[56]. One of the few known bacterial P450s that possesses a similar system is CYP102 from *Bacillus megaterium*, where the reductase moiety is fused with the hemoprotein domain[57]. Although the prokaryotic origins of mitochondrial P450s can be regarded as hypothetical[17], there is compelling evidence from mRNA comparison that support the theory[54,55].

In order for the P450-mediated reaction to take place it is necessary for there to be two consecutive reduction steps, whereby the electronic state of bound dioxygen is successively altered by the addition of two electrons from reductant (NADH or NADPH), a process which is carried out by a P450-bound redox partner (i.e., redoxin or flavoprotein reductase). In order for this two-stage reduction to occur, however, the dioxygen molecule is required to constitute the terminal species of an electron transfer gradient[45] formed by the sequential stages outlined below:

NAD(P)H $\rightarrow$ FAD $\rightarrow$ FMN $\rightarrow$ P450
in the microsomal system (and CYP102) or
NADH $\rightarrow$ FAD $\rightarrow$ $Fe_2S_2$ redoxin $\rightarrow$ P450
in mitochondrial and bacterial systems where the arrow ($\rightarrow$) represents the transfer of a single electron.

Consequently, the redox potentials of each component in the electron transport pathway should become less negative as one proceeds down the electron transfer chain[45]. Table 4 shows the standard redox potentials ($E^o$ values) for the participant species in the bacterial $P450_{cam}$ (CYP101) system, together with those of the relevant dioxygen couples[2]. This table indicates that, in its resting state, $P450_{cam}$ has a too highly negative redox potential (-303 mV) to become reduced by its redox partner, putidaredoxin. However, when the substrate camphor binds to $P450_{cam}$, there is a dramatic lowering of the redox potential (i.e., it becomes less negative) which becomes -173 mV in the substrate-bound enzyme[52]. In this way, substrate binding is able to control the P450 catalytic cycle by facilitating reduction of the enzyme[2,45].

Additionally, the iron spin-state equilibrium is significantly altered when the substrates binds, and it appears that there is substantial coupling between spin and redox equilibria in both

**TABLE 4**
**Redox Potentials ($E^o$) in the $P450_{cam}$ System[2]**

| Species | $E^o$ (mV) |
|---|---|
| NADH | -320 |
| FAD | -290 |
| Pdx | -240 |
| $P450_{cam}$ | -303 |
| $P450_{cam}$ + substrate | -173 |
| $O_2/O_2^-$ | -160 |
| $O_2^-/O_2H$ | -100 |

*Abbreviations*: NADH = nicotinamide adenine dinucleotide; FAD = flavin adenine dinucleotide; Pdx = putidaredoxin; $P450_{cam}$ = camphor 5-exo monooxygenase in *pseudomonas putida.*

the bacterial and microsomal P450 systems[58]. Table 5 indicates that substrate binding modulates the iron spin-state equilibrium with concomitant lowering of redox potential in $P450_{cam}$ (CYP101) and also in rat liver microsomal P450, where there is strong correlation (R = 0.96) between % high-spin and iron redox potential[2]. In fact, there is a clear relationship between shift to high-spin for the heme iron in microsomal P450 and rate of metabolism of benz-phetamine analogues[59], thus indicating that substrate binding triggers the entire P450 catalytic cycle by modulating the spin-state equilibrium, lowering the P450 $Fe^{3+}/Fe^{2+}$ redox potential and thus facilitating reduction of the heme iron from ferric to ferrous, such that dioxygen is able to bind to the reduced P450 and, consequently, become activated in order to oxygenate the substrate[45,56]. It is important, therefore, to appreciate why the P450 spin-state shift is essential for catalytic activity.

If one considers the thermodynamics of a typical P450 reaction, it is clear that the products are of lower energy than the reactants, but the uncatalyzed activation energy of the process (estimated to be 418–460 $kJ.mol^{-1}$) ensures that the reaction is kinetically unfavourable at normal physiological temperatures[60]. However, the enzyme lowers this activation energy (to between 38–71 $kJ.mol^{-1}$) by binding the substrate close to the heme group and by binding oxygen at the heme iron which, via the influence of the invariant cysteine, becomes activated following electron transfer[2].

A key prerequisite for any organic reaction is that the reactants are in the same spin-state. Most organic compounds are in the singlet state, with paired electron spins, whereas molecular oxygen is in the triplet ground state with two impaired electrons[61]. Consequently, the reaction between molecular dioxygen and organic substrates is termed spin-forbidden at normal temperatures[61]. Cytochrome P450 is able to catalyze this reaction, however, by altering the electronic state of oxygen, but the enzyme also makes use of the triplet ground state to ensure a high binding affinity between oxygen and the heme iron which, in the presence of substrate, is also in a high-spin state (see Table 5). Furthermore, the oxygen binding affinity of hemoproteins in general is proportional to their iron redox potential[62] and, therefore, the substrate-dependent increase in $E^o$ for the $Fe^{3+}/Fe^{2+}$ couple (i.e., it becomes less negative) is likely to be an additional factor in explaining the high oxygen binding affinity ($K_D$ = 0.6 µM in CYP101) of substrate-bound reduced P450.

Consequently, the reaction between the heme iron and molecular oxygen in the P450 system is energetically favourable provided that the iron is in the high-spin state and is reduced from $Fe^{3+}$ to $Fe^{2+}$, because this places an overall negative charge on the iron porphyrin system which is then transferred to the electron-deficient dioxygen molecule[63]. Moreover, the resulting activation of oxygen by the successive addition of two reducing equivalents ($2H^+$, $2e^-$) gives rise to a singlet state peroxide species[64] that readily reacts with a singlet state substrate bound in the immediate vicinity of the heme iron-oxygen complex.

Energetically, the consecutive donation of two electrons to dioxygen brings about a lengthening (and, hence, weakening) of the oxygen-oxygen bond, as shown in Table 6. Therefore, the P450-catalyzed activation of oxygen can be represented as follows:

$$\underset{\text{dioxygen}}{O_2} \xrightarrow{e^-} \underset{\text{superoxide}}{O_2} \xrightarrow{e^-} \underset{\text{peroxide}}{O_2^{2-}}$$

In this way superoxide is produced following the electron transfer from reduced ($Fe^{2+}$) P450 to oxygen which is, in turn oxidized back to the $Fe^{3+}$ state[56]. The second reduction of P450 then facilitates the transfer of another electron from $Fe^{2+}$ to the superoxide anion such that the reactive peroxide species is formed[64].

**TABLE 5**
**Spin-State and Redox Potential ($E^o$) in P450s[8]**

| System | % High-Spin | $E^o$ (mV) |
|---|---|---|
| $P450_{cam}$ | 8 | -303 |
| $P450_{cam}$ + camphor | 94 | -173 |
| $P450_{rlm}$ | 10 | -300 |
| $P450_{rlm}$ + hexobarbital | 35 | -237 |
| $P450_{rlm}$ + benzphetamine | 38 | -225 |

% high-spin = 0.61$E^o$ + 187.1 (±0.11); correlation coefficient = 0.96.

$P450_{cam}$ = camphor 5-exo monooxygenase from *pseudomonas putida;* $P450_{rlm}$ = rat liver microsomal P450 isozymes.

Data compiled from Sligar et al., 1979[58].

The likely stages in the P450 catalytic cycle are represented schematically in Fig. 4, which shows how the heme system in P450 is able to direct the course of the reaction during substrate binding, reduction, oxygenation, and rearrangement of the oxygenated complex leading to the formation of products[2]. It would appear that the P450 system achieves oxygen activation and mono-oxygenation of substrates in a carefully controlled, subtle fashion which has evolved over at least 2000 million years ago since the earth's atmosphere has contained free oxygen[11]. Consequently, the P450 tertiary structure would have evolved (possibly like the globins) to bind:

1. heme, via the invariant cysteine residue, together with the cooperation of at least two basic amino acids residues which form ion-pairs with the negatively charged propionate side chains on the heme moiety;
2. oxygen, via a deformation in the distal I helix which includes conserved glycine and threonine residues, together with a conserved acidic residue which acts cooperatively with the threonine to facilitate proton transfer to the activated oxygen moiety;
3. substrates, via the spatial orientation of key amino acid residues in the vicinity of the heme pocket which, although being otherwise essentially hydrophobic in nature, also enables the 'fine-tuning' of substrate regio-specificity in metabolism by genetically 'engineering' changes in active site residues; and
4. redox partners, for the consecutive input of two electrons via the placement of surface basic residues close to the proximal heme face, where a nearby hydrophobic channel facilitates proton transfer by solvating water molecules[65] via a conserved acidic residue downstream from the invariant cysteine ligand.

Evolutionary constraints would also have to provide a means of regulating all of these above features (namely, 1 to 4) by placing the enzyme complex in a membrane phospholipid environment[45]. Moreover, the porphyrin ring system coordinating iron in P450 (and other hemoproteins) places the electronic state of the heme unit in an ideal situation for the binding of small molecular ligands in an environment capable of being modulated by the presence (or absence) of water molecules, which affect both the spin- and redox-states of the iron atom[5]. Furthermore, the axial heme ligands alter the 3d orbital energy level splitting such that resulting electronic transitions effect a spectral change in the UV absorption maximum which, in the reduced P450 carbon monoxide complex, is characteristically situated at 450 nm[66]. This intense absorption in the electronic spectrum led, of course, to the original discovery of the enzyme, together with its name[4]. The various stages in the P450 cycle will now be reviewed with respect to substrate binding, oxygen activation, and insertion such that the mechanism of oxygenation can be appreciated.

## STAGES IN THE P450 CATALYTIC CYCLE AND MECHANISM OF OXYGENATION

As mentioned previously, P450 activity is regulated by substrate binding as this gives rise to a change in the hemoprotein from low-spin to high-spin ferriheme, which brings about a lowering of the $Fe^{3+}/Fe^{2+}$ redox potential (moving from –303 mV to –173 mV in the bacterial system $P450_{cam}$). This facilitates electron transfer from either a redoxin or reductase redox partner, which is of redox potential around 290 mV[45]. The change from low- to high-spin $Fe^{3+}$ in P450 during substrate binding can be monitored spectroscopically[52] due to the fact that the alteration in electronic state of the heme iron affects the extent of 3d orbital splitting, leading to a shift in the Soret absorption maximum caused by a variation in electronic transitions within the cysteinate-heme moiety (reviewed in Reference 2).

Due to the differences between the ionic radii of the high- and low-spin iron, especially that of the $Fe^{3+}$ state, the iron atom will be expected to move out of the plane of the porphyrin ring in high-spin heme iron; this has been observed in the

**TABLE 6**
**Data for Oxygen Species[2]**

| Species | Bond Length (Å) | Bond Energy ($kJ.mol^{-1}$) | O–O Stretch ($cm^{-1}$) |
|---|---|---|---|
| $O_2$ | 1.21 | 497 | 1555 |
| $O_2^-$ | 1.33 | 276 | 1107 |
| $O_2^{2-}$ | 1.49 | 146 | 850 |

NB: The uncatalyzed activation energy for typical P450-mediated reactions lies in the range 418–460 $kJ.mol^{-1}$ which is close to the bond energy value for molecular oxygen.

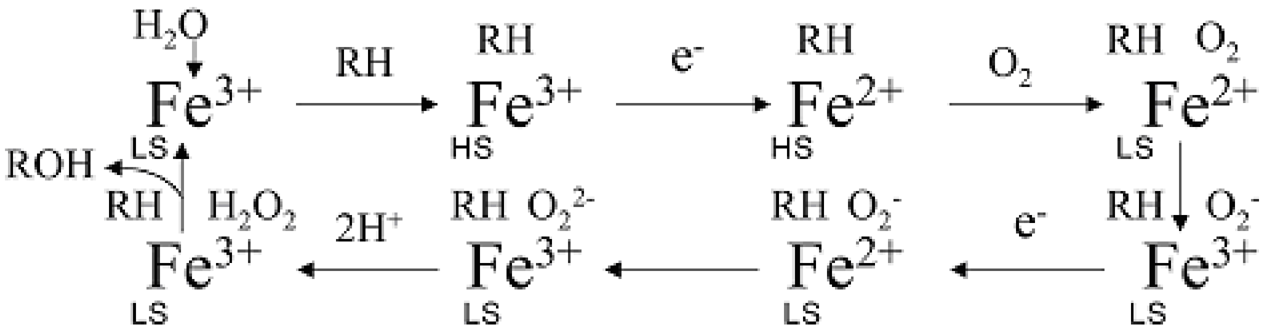

**FIGURE 4.** A proposed scheme for the P450 catalytic cycle (adapted from Reference 2). RH = hydrocarbon substrate; ROH = oxygenated metabolite; HS = high-spin; LS = low-spin.

crystal structure of substrate-bound $P450_{cam}$[67]. Table 7 compares the iron-sulphur and iron-nitrogen distances in substrate-bound and substrate-free $P450_{cam}$ (CYP101) where there is a change from high-spin to low-spin ferric iron, respectively. The iron atom lies about 0.4Å out of the porphyrin ring plane, and the difference between high- and low-spin $Fe^{3+}$ ionic radii is sufficient to account for the movement of iron toward the cysteinate sulphur atom in substrate-bound $P450_{cam}$ (see Table 7). It is likely that substrate binding brings about a conformational change in the P450 which is associated with substrate-induced desolvation of the heme environment[65] and movement of the iron atom out of the plane of the porphyrin ring. Although there are few differences in substate-bound and substrate-free $P450_{cam}$[6,67], comparison between substrate-bound and substrate-free $P450_{bm3}$ (CYP102) shows that significant conformational changes occur for the larger substrate, palmitoleic acid[68,69].

Following reduction of the substrate-bound P450 complex, there is evidence to suggest that the substrate moves closer to the heme iron[70] on the basis of NMR paramagnetic shift data[71]. It is possible that the subsequent increased negative charge on the heme moiety brings about an attraction of the substrate hydrogen atoms, which bear a partial positive charge, and it should be recalled that oxygenation usually proceeds via proton abstraction of the substrate[63]. The rate of initial reduction is substantially faster than the second reduction, which is generally regarded as being rate-limiting in both bacterial and microsomal systems[56]. The first reduction is probably triggered by the conformational change in the P450 apoprotein which accompanies substrate binding and, in the microsomal system, may relate to a reorientation of the P450 molecule within the membrane phospholipid such that interaction with reductase is facilitated[72].

When in the $Fe^{2+}$ state, oxygen binding occurs due to the higher oxygen affinity for the ferrous high-spin state, and this could proceed via spin-spin coupling between triplet ground state dioxygen (with two unpaired electrons) and high-spin $Fe^{2+}$ which contains four unpaired electrons (reviewed in Reference 2). As it is known that the interaction energy between two spin-free species is proportional to the product of their spin quantum numbers, the strong binding between reduced P450 and molecular dioxygen may be at least partially determined by the spin-pairing interaction, although the electron-deficient oxygen species will be attracted to the electron-rich $Fe^{2+}$ P450 and this could represent the dominant force in the oxygen binding process[5]. The ferrous iron in P450 changes to low-spin when oxygen is bound to the distal heme face, and the local environment of the P450 active site favours the electron transfer of $Fe^{2+}$ to oxygen, giving rise to the $Fe^{3+}$ $O_2^-$ state[56]. It is thought that the presence of protonated basic amino acid residues, and the likelihood of proton transfer pathway[65] via hydrogen-bonded conduits within the apoprotein, will lead to protonation of the resultant superoxide anion according to the process:

$$O_2 + H^+ \rightleftharpoons HO_2^{\cdot}$$

which possesses a high equilibrium constant ($K = 10^{12}$) such that the protonated superoxy species is favoured at normal temperature[73]. This, in turn, would have the effect of promoting the formation of iron(III)superoxide from the initial iron(II)dioxygen complex in P450[2,56].

The presence of superoxide in the $P450_{cam}$ system has been directly observed under catalytic conditions using reasonance Raman spectroscopy[74]. Table 6 indicates that the O-O stretching frequency is characteristic of the dioxygen species electronic state, and the oxygen-oxygen stretch in $P450_{cam}$ at 1141 $cm^{-1}$ appears to confirm the formation of an iron-bound superoxide[74]. In fact, the addition of superoxide to substrate-bound reduced $P450_{cam}$ is catalytically competent, and indicates that the initial $Fe^{2+}O_2^-$ complex may also undergo electron transfer to form an iron(III)peroxide species[75].

**TABLE 7**
**Interatomic Distances in the $P450_{cam}$ Heme Group and $Fe^{3+}$ Ionic Radii (Å)**

| Atomic Distance | $P450_{cam}$ (HS) | $P450_{cam}$ (LS) |
|---|---|---|
| Fe-S | 2.177 | 2.254 |
| Fe-N | 2.034 | 2.024 |
| Fe-$C_t$ | 0.433 | 0.0 |
| $Fe^{3+}$ radius | 0.64 | 0.55 |

$C_t$ is the centre of the porphyrin ring plane.

The shortening of Fe-S bond length in substrate-bound $P450_{cam}$ (0.08 Å) is about the same as the difference between the ionic radii (0.09 Å).

*References:* Crystal structure of substrate-bound $P450_{cam}$[67]; crystal structure of substrate-free $P450_{cam}$[6]; ionic radii of high-spin and low-spin $Fe^{3+}$[77].

This finding may provide a clue to the final stages in the P450 catalytic cycle where the oxygenated complex is reduced via a second electron transfer from the redox partner (reviewed in Reference 56). Based on the assumption that the initial reduced P450-dioxygen adduct tends to form an iron(III) superoxide via the process:

$$Fe^{2+}O_2 \rightleftharpoons Fe^{3+}O_2^{-}$$

then the second reduction would give rise to iron(II)$O_2^-$ by the input of another electron to iron(III)$O_2^-$[63]. The ferrous-superoxide complex is analogous to the situation produced artificially by the addition of superoxide to reduced $P450_{cam}$[75] and it is possible that, for the same reasons outlined previously for the iron(II)$O_2^-$ complex, there is a conversion to an iron(III)peroxide according to the process:

$$Fe^{2+}O_2^{-} \rightleftharpoons Fe^{3+}O_2^{2-}$$

Ferric-bound peroxide is highly reactive[64] and then breaks down, possibly under the influence of solvated protons in the heme environment, to oxygenate the bound substrate; this process, which is not fully understood, may involve the transient formation of a bare oxygen atom[4], probably produced via the decomposition of heme-bound hydrogen peroxide according to the reaction:

$$H_2O_2 \longrightarrow H_2O + [O$$

This sequence of stages is consistent with the production of water, which is also formed in P450 reactions, together with the mono-oxygenated metabolite resulting from single oxygen atom insertion into the substrate molecule[63]. It can be appreciated that the catalytic cycle (as outlined above) returns the P450 to its low-spin ferric resting state after having undergone the electronic changes described previously. There is clear experimental evidence for each intermediate proposed in the various stages of the P450-mediated oxygenation of organic substrates, apart from the high reactive single oxygen atom, although its presence has been reported in model systems[76]. However, it is apparent that the binding of substrate triggers the entire P450 catalytic cycle by bringing about a series of carefully controlled changes in the P450 system which leads to oxygen binding, activation and single oxygen insertion into the substrate, thus forming the mono-oxygenated product with the concomitant production of a water molecule (reviewed in Reference 2). Substrate binding appears to affect the haem iron spin-state equilibrium where, in general, there is a substrate-induced change from low-spin to high-spin iron (reviewed in Reference 2) and data from iron complexes[77] indicates that the iron is likely to move out of the heme plane, as is observed in the crystal state[67]. Moreover, substrate binding alters the hemoprotein redox potential, which may be related to the extent of heme exposure[78] and it is also possible that this has a bearing on the rate of substrate turnover. Table 8 provides details of the catalytic turnovers in selected P450 systems, with the CYP102 reaction proceeding at a rate close to the diffusion-controlled limit for an enzyme catalyzed reaction[7].

## CONCLUSIONS

P450 enzymes play an important role in the metabolism of a vast number and variety of both endogenous and exogenous chemicals[46]. Although their general reaction of mono-oxygenation is common to most P450 functions, they can catalyze the oxidative metabolism of virtually every class of organic chemical due to certain key complementary amino acid residue interactions within the active sites of different P450s[49] which govern their various substrate specificities, notwithstanding some degree of overlap in the mammalian P450s associated with xenobiotic metabolism.

The major roles of mammalian P450s include the following:

1. steroid hormone biosynthesis (CYP11, CYP17, CYP19, and CYP21);
2. activation of chemical carcinogens (CYP1 and CYP2E);
3. detoxication of foreign compounds (CYP1, CYP2, and CYP3);
4. metabolism of fatty acids, prostanoids, and eicosanoids (CYP4); and
5. development and maintenance of sex characteristics (CYP1, CYP2, and CYP3).

Other P450s present in fungi (CYP51-62), plants (CYP71-92), and bacteria (CYP101-118) are associated with the metabolism of natural products such as lanosterol (CYP51), citral (CYP71A5), and erythromycin (CYP107 and CYP113). Over 1200 P450 gene sequences have been determined from over 85 eukaryotic species and in excess of 20 prokaryotic species[1]. However, the three-dimensional structures of only 7 P450s have been characterized by X-ray crystallography and all of these are from bacterial sources[6,67,68,69,70]. Nevertheless, the tertiary fold is generally well conserved for all of these crystal structures despite the fact that the substrates vary from camphor (CYP101), palmitoleic acid (CYP102), a-terpineol (CYP108), and erythromycin (CYP107). Comparison of P450 gene and protein sequences indicate that the entire gene superfamily evolved over a period of at least 2000 million years, and construction of the P450 phylogenetic tree provides a number of interesting concordances with the generally accepted time-course for the evolution of biological systems on earth[11]. There is evidence to suggest that the gradual rise of atmospheric oxygen levels since the atmosphere changed from being reducing to oxidizing in nature over the last 2000 years (brought about by the advent of photosynthesizing bacterial species), played a key role[18,25,26] in the evolution of eukaryotic species, including: vertebrates, mammalia, primates, and, eventually, *Homo sapiens*. However, the possibility of variable rates of P450 evolution over time can lead to difficulties in making accurate comparisons with biological development over large time periods.

In addition to the likelihood that the increase in atmospheric oxygenation drives evolutionary change, where the P450 isozymes have played a significant role in both steroid hormone biosynthesis and xenobiotic metabolism, for example, it is possible that the pattern of global mass extinctions over geological time can be linked with terrestrial cycles of supercontinental fission and fusion events, together with planetary orbital periods and galactic rotation of the solar system[27,29,30]. Among other global changes produced via these cyclical effects, the rise in eustatic sea level appear to coincide with a number of major marine extinctions over the past 500 million years or more[29]. Consequently, a combination of first and second order terrestrial cycles probably accounts for the majority of global biological extinctions, which led to the dominance of mammalian species in recent geological time (i.e., since the last mass extinction occurred 65 million years ago). The current complement of drug-metabolizing P450s in man and other mammalian species may have originally developed as a response to plant toxins biosynthesized to deter animal predators when land colonization began around 400 million years ago[8,10]. It is, therefore, a fortunate circumstance that modern drugs have been designed largely from plant alkaloid templates, because the necessary detoxifying enzymes are already present for drug metabolism in man.

There are exciting possibilities for utilizing a bacterial P450 such as CYP102 in biotechnological applications such as bioremediation and biodegradation[79]. The CYP101 enzyme has already been employed in similar ways[80] for catalysing the aromatic hydroxylation of diphenylmethane, whereas its usual function is camphor-5-exo hydroxylation. Consequently, only

**TABLE 8**
**Catalytic Turnover Numbers for Different P450s**

| P450 | Turnover ($min^{-1}$) | Substrate | Type | Redox Partner | Class of P450 |
|---|---|---|---|---|---|
| CYP2B4 | 29 | Benzphetamine | Microsomal | Reductase | II (E)* |
| CYP101 | 1900 | Camphor | Bacterial | Redoxin | I (B) |
| CYP102 | 4600 | Lauric acid | Bacterial | Linked reductase | II (E) |

* E = eukaryotic; B = bacterial.

*References:* information compiled from References 2, 48, and 56.

a single mutation can give rise to a genetically engineered enzyme with markedly different catalytic selectivity[80]. Applying this principle to CYP102 would be advantageous as this fused enzyme exhibits catalytic self-sufficiency coupled with a significantly higher turnover rate[57].

## ACKNOWLEDGEMENTS

The financial support of GlaxoWellcome Research & Development Limited, Merck, Sharp & Dohme Limited, the European Commission Biomed II programme (EUROCYP) and the University of Surrey Foundation Fund is gratefully acknowledged by one of us (DFVL).

| | | |
|---|---|---|
| Received: | December | 29, 2000 |
| Revised: | March | 15, 2001 |
| Accepted: | March | 16, 2001 |
| Published: | April | 19, 2001 |
